# SMPDL3B contributes to gastric adenocarcinoma cells progression by promoting the infiltration of M2 macrophages

**DOI:** 10.55730/1300-0144.5732

**Published:** 2023-10-04

**Authors:** Li SU, Jian HAO, Na ZHANG, Shan WU, Xiuhua WU, Wei WEI

**Affiliations:** 1 Department of Rheumatology and Immunology, Tianjin Medical University General Hospital, Tianjin Medical University, Tianjin, China; 2 Department of Oncology, Tianjin Union Medical Center, Nankai University, Tianjin, China

**Keywords:** Gastric adenocarcinoma, SMPDL3B, tumor progression, immune evaluation

## Abstract

**Background/aim:**

The common disease gastric adenocarcinoma (GAC) has a high morbidity and mortality, so there is an urgent need for research to explore new diagnostic markers and therapeutic targets. This investigation was carried out to investigate the expression of sphingomyelin phosphodiesterase acid-like 3b (SMPDL3B) in GAC and its effects on tumor progression.

**Materials and methods:**

Samples were collected from patients who underwent radical gastrectomy from January 2021 to December 2022. Along with the normal gastric epithelial cell lines GES-1 and SGC-7901, the AGS, MGC-803, and MSN-45 human gastric cancer cell lines were used to confirm SMPDL3B expression. RT-qPCR, Western blot, immunohistochemical, cell proliferation, assay of wound healing, transwell migration assay, invasion assay, flow cytometry, and immune evaluation experiments were carried out.

**Results:**

SMPDL3B was found to be substantially expressed in GAC, and this condition has a bad prognosis. By establishing SMPDL3B knockdown and overexpression of GAC cell lines, this study confirmed that SMPDL3B promoted tumor cell proliferation, migration, and invasion. Additional bioinformatics research revealed a connection between SMPDL3B and immune cell infiltration in the GAC immunological microenvironment, which enhanced tumor cell proliferation by promoting the infiltration content of M2 macrophages.

**Conclusion:**

This study determined the function of SMPDL3B for the clinical diagnosis, prediction, and novel management of GAC.

## 1. Introduction

Stomach and gastroesophageal junction adenocarcinoma is the fourth most prevalent cancer globally and has a significant prevalence [1,2]. With an estimated 36%–75% 5-year survival rate, the prognosis for the illness is not promising [3]. The high-risk regions for gastric adenocarcinoma (GAC) incidence and death are East Asia, Eastern Europe, and South America, with the incidence of stomach cancer being twice as high in men as in women [4]. Meanwhile, GAC also has a high disease burden in China, according to a 2020 report [5]. The high morbidity and mortality of this disease has greatly afflicted people all over the world.

The diagnosis and treatment of GAC have advanced remarkably; however, despite significant advancements in surgery, radiation, and chemotherapy, the disease’s survival rate is still exceedingly poor [6,7]. From the perspective of clinical treatment, surgical excision is still the main method to treat the early stages of GAC [8]. It has been reported that understanding the molecular mechanism of the biogenesis and progression of GAC is crucial for the development of more effective therapeutic approaches beyond surgical treatment [9]. Thus, how to improve the diagnostic efficacy of GAC or develop new targeted therapeutic strategies through some important molecular targets has become an important aspect in the research of this disease.

According to reports, sphingomyelin phosphodiesterase acid-like 3b (SMPDL3B) is critical for maintaining the fluidity of cell membranes and controlling their lipid levels [10,11]. One of the most extensively researched elements of this molecule is its crucial function in the kidney. The overexpression of SMPDL3B protects versus radiation-induced changes in nuclear ceramide-1-phosphate (C1P) and ceramide levels, which has been linked to a unique role for the protein in DNA damage responses [12]. SMPDL3B has been shown to be a viable therapeutic target for various disorders and plays a significant role in the onset and progression of a number of diseases. It has been shown that the high expression of SMPDL3B in diabetic kidney disease podocytes affects the production of active sphingolipids [11]. The control of radiation-induced harm to human glomerular endothelial cells and renal podocytes is another important function of SMPDL3B [13,14].

The effects of SMPDL3B have also been demonstrated in some tumor diseases. SMPDL3B has been proposed as a possible therapeutic target and an efficient predictive biomarker for acute myeloid leukemia (AML) [15]. In prostate cancer tissues, Frank et al. [16] discovered that SMPLD3B was substantially overexpressed, which was adversely correlated with the prognosis of local prostate malignancy and knockdown. The prostate cancer cells’ migration was inhibited by SMPDL3B. A recent study based on whole exon sequencing also found that neoantigen-reactive T cells derived from colorectal cancer patients and containing SMPDL3B-T452M mutations showed a higher antitumor response than controls [17].

However, the expression of SMPDL3B in GAC and its effect on GAC cells have not been discussed before. In order to determine the role that this molecular expression plays in the prognosis of GAC, this study was designed to examine the expression of SMPDL3B in several tumor tissues, including GAC. Additionally, by creating GAC cell lines with SMPDL3B knockdown and overexpression, we will examine the impact of the SMPDL3B gene on tumor cell activity and its potential mechanism.

## 2. Methods and materials

### 2.1 Data acquisition and bioinformatics analysis

The online bioinformatics analysis was based on information from common websites and datasets derived from publicly available sources. The Cancer Genome Atlas Stomach Adenocarcinoma (TCGA-STAD) database was also used to analyze the expression of SMPDL3B in pancancer and various stages of GAC using UALCAN (https://ualcan.path.uab.edu) [18]. To specifically examine SMPDL3B expression in GAC and its survival prognosis based on TCGA-STAD datasets, GEPIA2 (http://gepia2.cancer-pku.cn/#index) was employed [19]. The Mantel–Cox test, which includes Cox proportional hazard ratios, 95% confidence intervals, and p values, was used to perform a survival analysis at the same website.

### 2.2 Tissue acquisition and processing

The clinical tissue samples used in this study were from 40 patients who underwent radical gastrectomy at the Tianjin Union Medical Center from January 2021 to December 2022. All tumor and normal tissue specimens were obtained through surgical operations in our hospital. There is a one-to-one correspondence between each tumor and its normal counterpart. Following the procedure, the specimens were taken away and immediately placed in liquid nitrogen before being stored at −80 °C for long-term continued use as needed. The individual’s informed permission was acquired for every sample collection, and all procedures were carried out according to the Declaration of Helsinki. The GAC and matching normal tissues were used for reverse transcription-quantitative polymerase chain reaction (RT-qPCR), immunofluorescence (IF), and immunohistochemical (IHC) examination. The Ethics Committee of the Tianjin Medical University General Hospital gave its approval to carry out this research (approval number: 3928/GAC/CH/2021).

### 2.3 Cell cultures

The SGC-7901, AGS, MGC-803, and MSN-45 human gastric cancer cell lines, as well as the normal gastric epithelial cell line GES-1, were employed in this investigation to confirm SMPDL3B expression. All cell lines were kept in our laboratory. All cells were grown in high-glucose Dulbecco’s modified Eagle medium (DMEM, Corning 10-013-CV) supplemented with 10% fetal bovine serum (Hyclone, FBS) and 1% penicillin/streptomycin solution (Gibco, P/S) at 37 °C and 5% CO_2_ (Thermo Fisher, US, 51023126). To obtain M2-conditioned media incubated with cancer cells, 150 μL of culture supernatant was mixed with 130 μL of water and 20 μL of Griess reagent, which was made fresh by mixing equal volumes of 0.1% N-(1-naphthyl) ethylenediamine dihydrochloride and 1% sulfanilic acid.

### 2.4 RT-qPCR

Trizol (Takara Bio, Inc.) was used to extract the total RNA from the cells and tissues. After chloroform separation, isopropyl alcohol precipitation, washing with 75% ethanol, and drying by air, H_2_O was used to suspend the RNA; the concentration was determined using the nanodrop method and stored at −80 °C. Using a reverse transcription kit (MCE, China, HY-K0510A), reverse transcription was carried out with 1 μg RNA. Primers using SYBR Premix Ex Taq (Takara Bio, Inc.), cDNA, and the genes under test were qPCR analyzed using the following thermal cycle conditions: 30 s of initial denaturation at 95 °C, 50 cycles of 5 s each of annealing and extension, followed by 30 s at 60 °C. All target gene transcripts were normalized to glyceraldehyde-3-phosphate dehydrogenase (GAPDH), and the relative fold change in expression was calculated using the 2^−ΔΔCT^ method.

The primers that were used have the following sequences:

SMPDL3B forward: 5′-GCGTCATAGCAGGGCAGTTCTT-3′
SMPDL3B reverse: 5′-TCCAGGTGTGATGAACATGGCG-3′
P21 forward: 5′-AGGTGGACCTGGAGACTCTCAG-3′
P21 reverse: 5′-TCCTCTTGGAGAAGATCAGCCG-3′
P16 forward: 5′-CTCGTGCTGATGCTACTGAGGA-3′
P16 reverse: 5′-GGTCGGCGCAGTTGGGCTCC-3′
CDK4 forward: 5′-CTCGTGCTGATGCTACTGAGGA-3′
CDK4 reverse: 5′-GGTCGGCGCAGTTGGGCTCC-3′
E-cadherin forward: 5′-GCCTCCTGAAAAGAGAGTGGAAG-3′
E-cadherin reverse: 5′-TGGCAGTGTCTCTCCAAATCCG-3′
CDK4 forward: 5′-CTCGTGCTGATGCTACTGAGGA-3′
CDK4 reverse: 5′-GGTCGGCGCAGTTGGGCTCC-3′
ZO-1 forward: 5′-GTCCAGAATCTCGGAAAAGTGCC-3′
ZO-1 reverse: 5′-CTTTCAGCGCACCATACCAACC-3′
Vimentin forward: 5′-AGGCAAAGCAGGAGTCCACTGA-3′
Vimentin reverse: 5′-ATCTGGCGTTCCAGGGACTCAT-3′
MMP-9 forward: 5′-GCCACTACTGTGCCTTTGAGTC-3′
MMP-9 reverse: 5′-CCCTCAGAGAATCGCCAGTACT-3′
CD206 forward: 5′-TGGCAGTGTCTCTCCAAATCCG-3′
CD206 reverse: 5′-CTCGTGCTGATGCTACTGAGGA-3′

### 2.5 Western blot (WB)

RIPA lysis buffer (MCE, China, HY-K1001) was used to extract the total protein from cells and tissues. Then, a BCA protein assay kit (Beyotime, China, P0012) was used to determine the protein concentration. After protein quantification, a concentrated gel and a separation gel were routinely prepared. SDS-PAGE was used to separate the protein samples (20 g per group), which were then transferred to 0.22 μm polyvinylidene difluoride (PVDF) membranes while maintaining a steady current. The PVDF membrane was incubated with the matching primary antibody (1:1000) at 4 °C for 12 to 16 h after being stored in a sealed container with 5% skim milk powder at room temperature for 2 h. The primary antibody information was SMPDL3B (Proteintech, China, 16552-1-AP) and GAPDH (Proteintech, China, 60004-1-Ig). Affinipure goat antirabbit IgG (H + L) (1:10000; Proteintech, China, SA00001-2) was HRP-conjugated after three PBS-Tween washings. It was then incubated at an ambient temperature for 2 h. The internal reference protein is GAPDH. The BeyoECL Moon Ultra-Sensitive ECL Chemiluminescence Kit (Beyotime, China, P0018FM) was used for band imaging. For additional grey value analysis, ImageJ (National Institutes of Health, US; version 1.45) was used.

### 2.6 IHC and IF staining

Frozen sections were used for the IHC and IF staining of the tumor and normal tissues. The sections were fixed for 25 min at an ambient temperature in a 4% paraformaldehyde (PFA) solution. To thoroughly remove the PFA, they were washed 3 times for 5 min each with phosphate-buffered saline (PBS). The segment was then treated with PBS containing 0.4% Triton-X for 20 min, followed by 1 h of blocking with a 5% bovine serum albumin solution. After the subsequent block, the diluted first antibody was incubated at 4 °C for 12–16 h for labeling. The primary antibody information was SMPDL3B (Proteintech, China, 16552-1-AP, 1:200) and CD206 (Proteintech, China, 60143-1-Ig, 1:400). The unbound antibody was washed 3 times in PBS, and the sample was incubated with the secondary antibody with constant shaking at room temperature for 2 h (IF requires shading). IF was used at a 1:400 dilution using secondary antibodies conjured with Alexa fluorescent dye (Molecular Probe). Antirabbit Alexa Fluor 488 and antirat Alexa Fluor 549 were used for green and red labeling, respectively. The unbound antibody was removed by washing 3 times with PBS at room temperature, and a cover glass was put on after adding the fluorescent stain DAPI. The IHC and IF were photographed using a stereomicroscope (Leica, Germany, S9) and a fluorescence microscope (Leica, Germany, DM2000), respectively, and images were obtained at 200× magnification.

### 2.7 Construction of overexpression and knockdown SMPDL3B cell lines

The AGS cell lines overexpressing SMPDL3B were obtained by the plasmids expressing SMPDL3B (pC-SMPDL3B) (GENECHEM, China), and empty Vector (Pc-DNA 3.1) was a negative control (GENECHEM, China). SMPDL3B knockdown MKN-45 cell lines were achieved with SMPDL3B-targeting (siRNA1 and siRNA2) with empty small interfering RNA (siNC) as a negative control. Pc-DNA and siRNA were transfected into the cells using Lipofectamine 3000 (Invitrogen, US, L3000001).

### 2.8 Cell proliferation

The MTT and colony formation assays were used to evaluate cell growth. For the MTT assay, 2000 cells/well, seeded onto 96-well plates, were grown for 4 days at 37 °C with 5% CO_2_. The cells were treated with MTT dye (Beyotime, China, C0009S) at 37 °C for 2 h, at which point the clock was reset to 0 h. The cell culture medium was removed and 150 mL dimethyl sulfoxide (DMSO, Beyotime, China, ST1276) was used to dissolve purple formaldehyde crystals. Absorbance was then measured at 570nm. The same process was carried out 24, 48, and 72 h later. The formula cell proliferation rate (%) = Tn/T0×100% was used to get the cell proliferation rate. For the colony formation assay, colonies were created by continually cultivating 1000 cells per well for 14 days in 6-well plates. The cells were stained with 1% crystal violet in anhydrous ethanol after being cultured and subsequently rinsed with PBS. They are then examined under a microscope, captured on camera, and numbered (Leica, Germany, S9).

### 2.9 Assay of wound healing

For this assay, the cells were implanted 3 days before the experiment, and the experiment was started when the cell fusion degree reached 100%. A 200-μL pipette tip was used to scrape the adhering cells onto a 6-well plate, which were then washed twice with PBS before being grown in media containing 1% FBS. Images were collected at 0, 24, and 48 h to determine the percentage of wound closure.

### 2.10 Transwell migration and invasion assay

A total of 1 × 10^5^ cells were planted in the top well of a 0.4 μm pore-size 24-well Transwell chamber (Thermo Fisher, America, 140620), which was either precoated with Matrigel matrix (invasion) or not (migration) (Corning, 354234). Then, 500 μL of 10% FBS total medium was poured into the bottom well chamber. The cells were rinsed with PBS and stained with 1% crystal violet in anhydrous ethanol following a 24-h incubation at 37 °C and 5% CO2. Afterwards, they were observed under a microscope, photographed, and counted (Leica, Germany, S9).

### 2.11 Flow cytometry

The cell cycle was evaluated using flow cytometry. The cells were cultivated for 1 week on 6-well plates, as described in section 2.3. After the media was removed, the cells were separated using trypsin, the digestion was stopped, and the cells were centrifuged at 12000 rpm for 10 min. The cell cycle analysis of the derived cells was based on DNA content. The regimen consisted of first cell fixation and then propyl iodide (PI, 500 μg/mL) staining. The cells were treated with a BD Cytofix/Cytoperm™ Kit (BD, US, 554714) after being washed twice with BD wash buffer for 30min. After centrifugation at 1500 rpm for 4 min, each cell sample was suspended in 500 μL staining solution. The staining solution contained RNA enzyme A (2 μL, Thermo Fisher Scientific, US), PI (20 μL, Thermo Fisher Scientific, US), Tween 20 (0.5 μL), and PBS (477.5 μL). The cells were examined using cytoFLEX flow cytometry (Beckman Coulter, US, B53000) after 30 min of incubation and fluorescence emission at 530 nm and 575 nm (or equivalent) was measured using 488 nm excitation.

### 2.12 Immune evaluation

The TCGA data were obtained in the manner previously mentioned [19]. Twenty-two samples of all the immune cells in the dataset were predicted using immune cell infiltration and tumor immunity analysis using the CIBERSORTx analytical tool (http://cibersortx.stanford.edu) and a SMPDL3B feature gene matrix. From the TCGA-STAD dataset, the proportions of the 22 different types of immune cells were assessed using CIBERSORTx, and the correlation between these 22 immune cell types was estimated. The connection among gene expression and these immune-infiltrating cells was computed by integrating candidate gene expression; p ≤ 0.05 indicates statistical significance. Key gene selection for immune checkpoints has been reported in the literature. Immunization scores for each sample in TCGA-STAD were obtained from the online TCIA database TCIA (https://tcia.at/home).

### 2.13 Statistical analysis

In this study, R-Studio (version 1.4) and GraphPad Prism (version 9.2) were used for statistical analysis. An unpaired t-test was used to compare the expression levels of the two groups. A conclusion was deemed statistically meaningful at p < 0.05. NS is p ≥ 0.05; * is p < 0.05; ** is p < 0.01; *** is p < 0.001; **** is p < 0.001.

## 3. Results

### 3.1 SMPDL3B is significantly expressed in GAC, and the degree of expression is connected with the disease’s prognosis and rate of development

**Figure 1:**
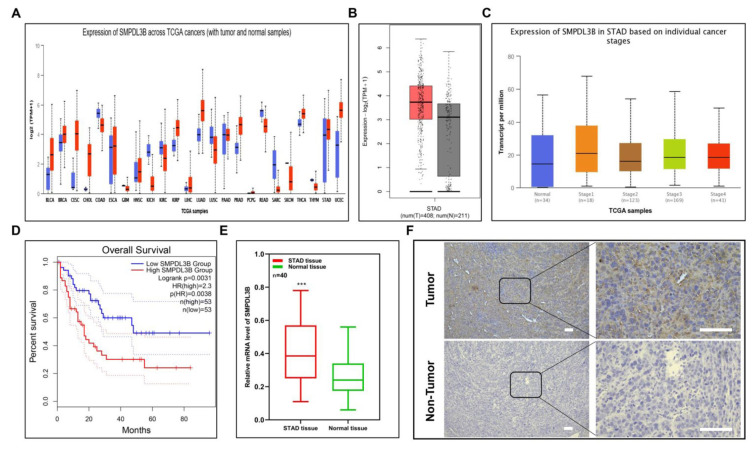
Differential expression of SMPDL3B in pancancer and the low expression of SMPDL3B in STAD is associated with prognosis. (a) Differential expression of SMPDL3B in 23 kinds of tumors and paired normal tissues in the TCGA database. (b) Expression of SMPDL3B in 408 gastric cancer samples and 211 normal tissues in the TCGA and GTEx databases. (c) Differential expression of SMPDL3B in normal tissues and different clinical stages of gastric cancer. (d) Relationship between SMPDL3B expression level and overall survival rate in patients with gastric cancer (cutoff = 50%, blue = low expression of SMPDL3B, red = high expression of SMPDL3B). (e) Differential expression of SMPDL3B in 40 pairs of clinical tissues (red = gastric cancer; green = normal stomach tissue). (f) Expression of SMPDL3B protein in representative tissue samples. Bar = 50 μm; *** is p < 0.001.

Initially, the impact of SMPDL3B on 23 tumors using the TCGA open dataset was investigated (BLCA, BRCA, CESC, CHOL, ESCA, GBM, HNSC, KICH, KIRC, KIRP, LIHC, LUAD, LUSC, PAAD, PRAD, PCPG, READ, SARC, SKCM, THCA, THYM, STAD, UCEC) (Figure 1a). The results show that SMPDL3B has a tendency for high expression in a variety of tumors, among which GAC was prominent. The GEPIA2 online analysis tool was used to discover that 408 GAC tissues had substantially higher levels of SMPDL3B expression than 211 normal gastric tissues from TCGA and GTEx sources (Figure 1b). Based on the TCGA-STAD data, our investigation results on the UALCAN online website showed that compared with normal tissues, GAC at different stages showed high expression of SMPDL3B, but with the progression of stages, the expression level did not change in a specific way (Figure 1c). According to the patient-prognostic information in the TCGA-STAD dataset, the overall survival of individuals with adenocarcinoma of the gastric region was also shown to be correlated with the differential expression of SMPDL3B, and patients with high expression of this gene had a worse prognosis (Figure 1d). In order to further confirm the authenticity of this conclusion, 40 GAC pairs of cases were used and paired with paracancer tissues obtained clinically in our center to study the differences in RNA (n = 40, Figure 1e) and protein (n = 2, Figure 1f) expression of SMPDL3B, and SMPDL3B was also shown to be substantially expressed in gastric cancer tissues, according to the findings.

### 3.2 Cell lines from stomach adenocarcinomas express increased SMPDL3B

**Figure 2:**
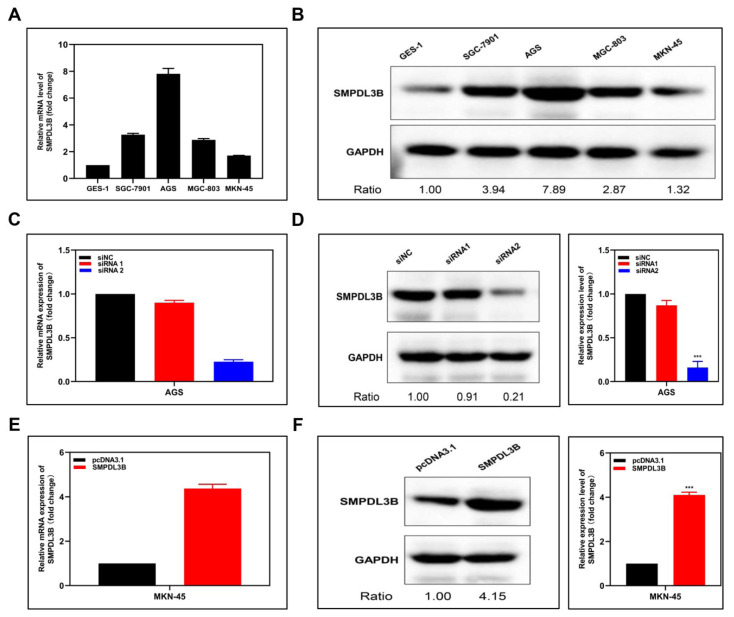
Expression and overexpression of SMPDL3B in different cell lines and construction of overexpression/knockdown cell lines. (a) mRNA expression of SPMPDL3B in normal gastric cell lines GES-1 and 4 gastric cancer cell lines. (b) Protein expression of SPMPDL3B in normal gastric cell lines GES-1 and 4 gastric cancer cell lines. (c,d) Expression of corresponding mRNA (c) and protein (d) in AGS cell lines after knockdown of SMPDL3B by 2 sequences of siRNAs. (e,f) Expression of corresponding mRNA (e) and protein (f) in MKN-45 cell lines after overexpression of SMPDL3B by pcDNA.

The normal gastric epithelial cell line GES-1 and the 4 gastric cancer cell lines SGC-7901, AGS, MGC-803, and MN-45 were found to express SMPDL3B mRNA (Figure 2a) and protein (Figure 2b). The findings demonstrate that, compared to healthy gastric epithelial cells, GAC cells expressed the SMPDL3B gene at a greater level. The greatest expression was seen in AGS cells, while the lowest expression was found in MKN-45 cells. Using the above conclusions, two different siRNA sequences were used to construct SMPDL3B-knockdown (KO) AGS cell lines, and the control group was treated with empty siNC. Through qPCR (Figure 2c) and WB (Figure 2d) verification, the siRNA2 treatment effect of our 2 showed better. In the same way, an overexpressed cell line based on the MKN-45 cell line was constructed using the pcDNA3.1 vector carrying SMPDL3B. The successful construction of the cell line was also confirmed by qPCR (Figure 2e) and WB (Figure 2f) verification.

### 3.3 SMPDL3B promotes the proliferation of adenocarcinoma gastric cells via influencing cell cycle progression

**Figure 3:**
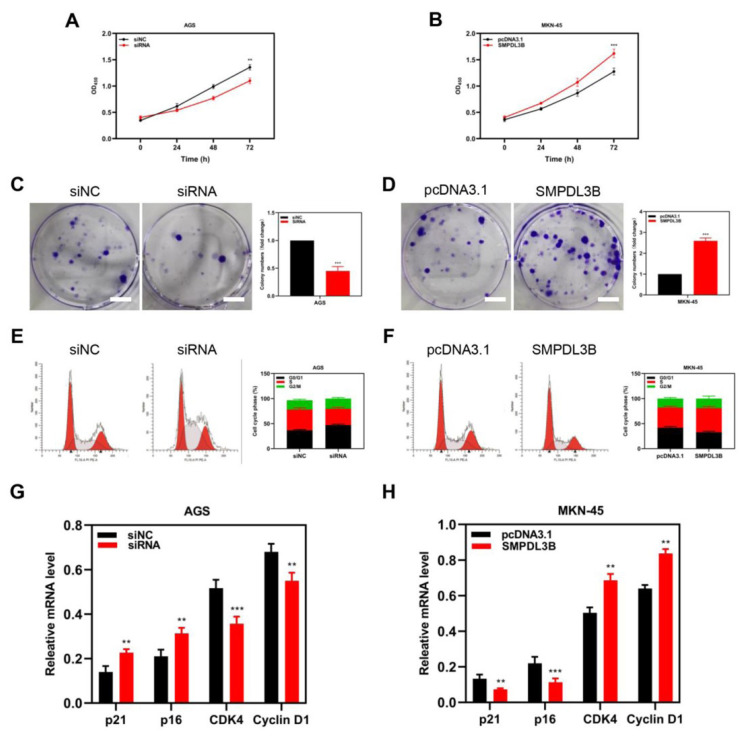
Proliferation phenotypic changes of tumor cell lines after SMPDL3B overexpression and knockdown. (a,b) The AGS cell line with SMPDL3B knockdown (a) and the MKN45 cell line with SMPDL3B overexpression (b) were compared with the parent cells by MTT assay. (c,d) Changes in the plate colony formation ability of tumor cell lines after knockdown (c) and overexpression (d) of SMPDL3B. (e,f) Knockdown (e) and overexpression (f) of SMPDL3B affect the cell cycle phase of STAD cells. (g,h) Differences in the expression of key proteins related to cell cycle regulation in tumor cells after knockdown (g) and overexpression (h) of SMPDL3B. Bar = 2 cm; *** is p < 0.001; ** is p < 0.01.

In the study, SMPDL3B overexpression or knockdown affected cancer cell growth and was investigated using the MTT cell proliferation assay. The findings demonstrated that SMPDL3B knockdown reduced the capacity of tumor cells to proliferate; in contrast, SMPDL3B overexpression increased the ability of tumor cells to proliferate (Figures 3a and 3b). Similarly, the effects of SMPDL3B changes on cell colony capacity were compared through a plate cloning formation assay. It revealed that the colonies formed by cells after SMPDL3B knockdown were only half of the previous amount (Figure 3c), while the numbers formed by overexpression were about 2.5 times the previous value (Figure 3d). The cell cycle analysis by flow cytometry showed that after SMPDL3B knockdown, fewer tumor cells were in the S phase and more of them were in the G0/G1 phase (Figure 3e). The number of cells in the S phase rose considerably after overexpressing SMPDL3B, but the number of cells in the G0/G1 phase dropped. (Figure 3f). The expressions of the key cell cycling-related proteins p21, p16, CDK4, and Cyclin D1 were detected before and after overexpression and knockdown. The results indicated that the expressions of p21 and p16 were increased after knockdown of SMPDL3B, while the expressions of CDK4 and Cyclin D1 were decreased (Figure 3g). After overexpression of SMPDL3B, the 4 molecules showed the opposite trend (Figure 3h).

### 3.4 SMPDL3B promotes the migration and invasion of GAC cells by affecting EMT-related proteins

**Figure 4:**
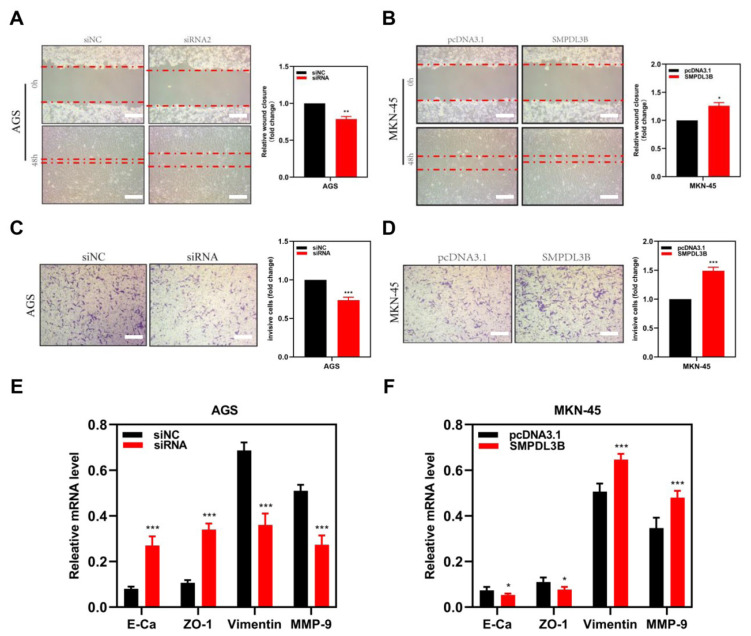
Migration phenotypic changes of tumor cell lines after SMPDL3B overexpression and knockdown. (a,b) The difference of scratch healing ability of tumor cells after knockdown (a) and overexpression (b) of SMPDL3B. (c,d) Transwell invasion assay was used to evaluate the difference in invasion and invasion ability of tumor cells after knockdown (c) and overexpression (d) of SMPDL3B. (e,f) Differences in the expression of key proteins related to migration and invasion regulation in tumor cells after knockdown (e) and overexpression (f) of SMPDL3B. Bar = 50 μm; *** is p < 0.001; ** is p < 0.01; * is P < 0.05.

This study investigated more closely how SMPDL3B affects the ability of gastric cancer cells to migrate and invade. The scratch recuperation process in GAC tissues greatly slowed down after this gene was knocked down, indicating that the capacity of GAC cells to migrate was weakened (Figure 4a). This was the first methodology used to investigate the impact of SMPDL3B on cell migration. However, after overexpressing SMPDL3B, gastric cancer cells had improved migratory abilities (Figure 4b) and it was further demonstrated through a transwell invasion assay that SMPDL3B knockdown reduced the capacity of gastric cancer cells to invade (Figure 4c). In contrast, the capacity of gastric cancer cells to invade was improved by overexpression of SMPDL3B (Figure 4d). These findings suggest that SMPDL3B is essential for gastric cancer cell invasion and migration. We investigated how SMPDL3B affects various key EMT proteins (E-cadherin, ZO-1, vimentin, and MMP-9) because epithelial-mesenchymal transformation (EMT) is a significant route of tumor cell migration and invasion. As a result of SMPDL3B knockdown, the expression levels of E-cadherin and ZO-1 rose, whereas those of vimentin and MMP-9 were considerably reduced (Figure 4e). Additionally, overexpression results in the reverse (Figure 4f).

### 3.5 In the tumor microenvironment of GAC, SMPDL3B plays an important role through immune regulation

**Figure 5:**
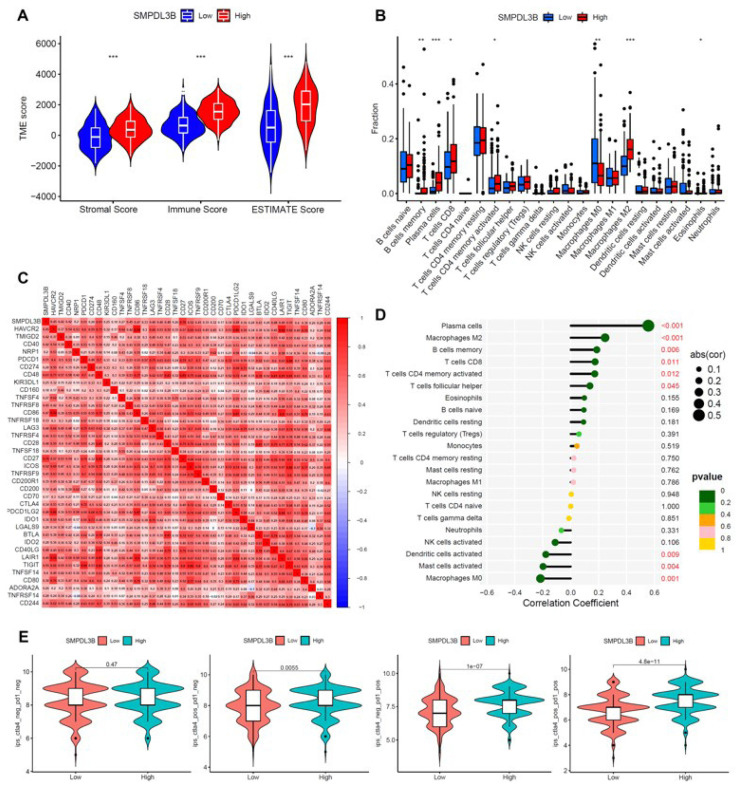
SMPDL3B expression is related to the regulation of immune microenvironment in GAC. (a) Difference in TME score between the two groups with high and low SMPDL3B expression (stromal score, immune score, and ESTIMATE score; cutoff = 50%). (b) Differences of key immune cells and immune molecules between the SMPDL3B high expression group and the SMPDL3B low expression group. (c) The correlation between SMPDL3B and important immune checkpoint molecules. (d) The correlation between SMPDL3B expression level and different immune cells. Circle size represents the correlation, and the positive and negative values on the horizontal axis respectively represent the positive and negative correlations. The relationship between p and color is shown in the bar. (e) The relationship between the PSMA6 high expression group and the low expression group and different combinations of antiPD1 or antiCTLA4 immunotherapy. ***is p < 0.001; ** is p < 0.01; * is p < 0.05.

The growth and origin of tumors is greatly impacted by immune-related control. As a result, the link among SMPDL3B-related genes and the tumor immune microenvironment (TME) of GAC was investigated, focusing on evaluating the relationship with key immune cells and immunotherapy target genes. For this purpose, tumor mRNA expression data from the TCGA-STAD were acquired and separated as 408 tumor samples into 2 groups: SMPDL3B-high and SMPDL3B-low, using the median expression of SMPDL3B as a cutoff value. The findings of our analysis of the TME-related scores of the two datasets revealed that the group with high SMPDL3B expression had greater immune scores (Figure 5a), suggesting that the high expression of SMPDL3B was related to positive immune regulation in GAC tissues. The relative contents of 22 different types of common tumor-infiltrating lymphocytes were then examined between the SMPDL3B high expression cohort and the low expression cohort. The findings revealed that the SMPDL3B high expression group had considerably greater relative numbers of memory B cells, activated CD4+ memory T cells, plasma cells, CD8+ T cells, M2 macrophages, and eosinophils. M0 macrophage levels, however, were less prevalent (Figure 5b). Next, the correlation between SMPDL3B and immune checkpoint-associated genes were identified. HAVCR2, TMIGD2, CD40, NRP1, PDCD1 and CD274 have previously been reported as immune checkpoint specific molecules. The expression of these genes and SMPDL3B were strongly linked (Figure 5c). M2 macrophages, activated plasma cells, CD8+ T cells, memory B cells, activated CD4+ memory T cells, and follicular helper T cells all showed positive correlations with PSMA6, whereas activated mast cells, M0 macrophages and activated dendritic cells showed the strongest negative correlation (Figure 5d). Next, the correlation among SMPDL3B and various tumor-infiltrating immune cells was further investigated. Ultimately, using the immunological score of 4 instances, the connection between immune checkpoint inhibitor combo treatments and SMPDL3B was investigated. The findings demonstrated that the immunological score of the SMPDL3B cohort with high expression was greater when CTLA4 and PD1 were combined, and the findings were highly significant. It is hypothesized that individuals with high SMPDL3B expression may benefit more from immunotherapy when combined with antiCTLA4+ antiPD1 (Figure 5e).

### 3.6 High expression of SMPDL3B promotes the infiltration of M2 macrophages in GAC tissues

**Figure 6:**
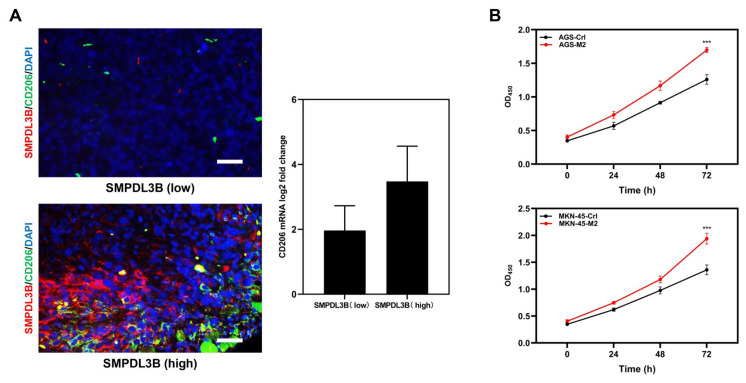
SMPDL3B affects the proliferation of GAC cells by affecting the content of M2-type macrophages. (a) The coexpression of SMPDL3B and M2-type macrophages in GAC was detected by immunofluorescence (red = SMPDL3B; green = CD206; blue = DAPI; bar = 50 μm). (b) Cell viability of GAC cells detected using the MTT assay after treatment with M2-conditioned medium or control medium. *** is p < 0.001.

Previously, it was observed that there is a substantial association between the number of M2 macrophages in the tumor microenvironment and the high expression of SMPDL3B, and this conclusion has been verified in the clinical samples. Through IF staining (Figure 6a) and qPCR (Figure 6b), it was determined that in GAC tissues with high expression of SMPDL3B, there were significantly more infiltrated M2 macrophages labelled with CD206. When cultivated in M2 conditioned media, gastric cancer cells proliferated at a greater rate, according to corresponding cell function assessments.

## 4. Discussion

As mentioned above, GAC has a high mortality and morbidity rate worldwide, and its heavy disease burden makes it a popular research topic. Early diagnosis and treatment of GAC can reduce disease-specific mortality [20,21]. Over the years, the prognosis of GAC has improved to some extent through the development of surgical treatment, chemotherapy, and targeted therapy [22,23]. However, the disease still has a poor prognosis. From the perspective of tumor therapy, the exploration of new target molecules for targeted therapy and diagnosis of GAC has been considered an important area of research. SMPDL3B is said to be involved in many tumors, according to findings in the literature, although investigations on this molecule in GAC have not been done.

The present study initially addressed how pancancer analysis revealed SMPDL3B expression in several cancer types and focused on the analysis of TCGA-STAD data to conclude that SMPDL3B showed a tendency for high expression in GAC tissues. Additionally, SMPDL3B was expressed at a greater level in various phases of GAC compared to normal tissues. Afterward, it was discovered through survival analysis that individuals with elevated SMPDL3B expression had worse clinical outcomes. By using clinical tissue samples and cell lines, the high expression of SMPDL3B was validated through the development of gastric adenoma cell lines that either overexpress or knockdown SMPDL3B. The outcomes indicate that SMPDL3B predominantly influences the control of the cell cycle as well as tumor cell invasion and migration through EMT-related proteins. The elevated expression of SMPDL3B was linked to the infiltration of M2 macrophages in the gastric cancer microenvironment, which could be established using the TCGA database and clinical samples.

According to several publications, SMPDL3B has a function in the growth and genesis of tumors. SMPDL3B has been proposed as a possible therapeutic target for AML and as a useful predictive biomarker for the disease [15]. The ability of hepatocellular carcinoma (HCC) cells to proliferate, migrate, and invade in vitro was established by Liu et al. [24], while knockout of SMPDL3B significantly inhibited HCC tumor formation in vivo. These results are consistent with our conclusions for GAC, confirming the cancer-promoting effect of SMPDL3B in different tumors. As for the mechanism, they found that ACER2 positively regulates SMPDL3B protein levels, and HCC cell viability is enhanced by ACER2/SMPDL3B’s promotion of ceramide hydrolysis and S1P synthesis [24]. According to Qu et al., human AML exhibits a significant upregulation of SMPDL3B expression. SMPDL3B is suggested as an independent prognostic predictor of AML due to the association between its high expression and poor overall survival. In terms of its functionality, the present research also revealed that reduced SMPDL3B expression may prevent AML cells from proliferating both in vitro and in vivo by encouraging the death of cells [15].

Additionally, Frank et al. [16] discovered that the overall survival of individuals with tumors receiving long-term follow-up was inversely correlated with SMPDL3B expression in cancerous prostate tissue samples taken from patients. Nevertheless, that study also found, using TCGA data, that both progression-free survival and recurrence-free survival were lower in patients with prostate cancer with low SMPDL3B expression. This result is not entirely in line with the findings made about stomach cancer. SMPDL3B may have various functions in different types of tumors and hence affect prognosis in varying ways. The specific reasons remain to be further explored.

It is worth noting that these studies, including ours, have shown that SMPDL3B can be greatly inhibited in its ability to function, either by knocking it down or eliminating it entirely. These findings imply that SMPDL3B may represent an essential new target for the treatment of tumors. The prognostic importance of SMPDL3B in various cancer types will be revealed by future research. Additionally, our work emphasized the connection between SMPDL3B and immunotherapy. SMPDL3B-related genes were strongly enriched in immune cell-related processes, such as granulocyte activation, neutrophilic activation, neutrophilic degranulation, and neutrophil-mediated immunity, according to the GO enrichment analysis of Qu et al. [15]. The infiltration of plasma cells and M2 macrophages in the tumor microenvironment, which included M2 macrophages and drew our interest for future investigation, was shown to be directly associated with the high expression of SMPDL3B, according to preliminary findings.

Macrophages are an important kind of immune cell in the human body. They play different functions by changing phenotype according to different environmental factors [25]. SMPDL3B-related genes were strongly enriched in immune cell-related processes, such as granulocyte activation. The M1 and M2 subtypes of activated macrophages are both tightly linked to the immune response but carry out different physiological roles. Proinflammatory responses are mostly mediated by M1 macrophages, whereas antiinflammatory responses are primarily mediated by M2 macrophages [25,26]. The majority of the macrophages in the tumor microenvironment are type M2, and studies have demonstrated that M2 macrophage infiltration might encourage tumor immune tolerance and speed up tumor growth [27–29]. Among macrophages, M2 macrophages usually play an antiinflammatory role in the tumor microenvironment, with poor antigen presentation ability, low IL-12, and high IL-10, IL-4, and IL-13 secretion characteristics, as well as immunosuppressive effects [25,30]. There has been some academic discussion about the relationship between SMPDL3B and macrophages. SMPDL3B-related genes are strongly enriched in immune cell-related processes, such as granulocyte activation. According to reports, SMPDL3B alters the cytolipid composition and membrane fluidity of macrophages by acting as a negative regulator of toll-like receptor signaling, thereby affecting macrophage function [31]. Taesik et al. found that macrophage-derived TSP1 inhibits the expression of SMPDL3B in the liver through an autocrine effect, increases the development of nonalcoholic fatty liver disease, and intensifies the liver’s proinflammatory signaling cascade [32]. In the tumor microenvironment, an abnormal inflammatory response is usually an important inducing factor for tumor cell proliferation, migration, and other functions [33]. Together, it is reasonable to hypothesize that SMPDL3B influences the polarization of macrophages in the tumor microenvironment and boosts the infiltration of M2 macrophages, which ultimately contributes to the poor prognosis of GAC.

Despite these gains in knowledge, there are still some limitations to our study. First, it has not shown evidence in vivo for the promotion of GAC cell proliferation by SMPDL3B. Secondly, the mechanism of SMPDL3B regulation on the biogenesis and development of GAC has not been clarified. To further explore the mechanism of SMPDL3B regulation, a more rigorous follow-up experimental design is needed, such as using RNA-seq to identify the key genes changed after overexpression or knockdown of SMPDL3B. The regulation mode of downstream molecules can be clarified by using protein interaction analysis. Ultimately, further clinical samples are required to validate our findings as well as determine the function of SMPDL3B in the clinical diagnosis, prediction, and novel management of GAC.

## 5. Conclusion

According to our findings, SMPDL3B is considerably overexpressed in GAC and is highly linked with its prognosis. High levels of SMPDL3B expression influence tumor cell proliferation through cell cycle, invasion and migration through the production of EMT-related proteins. SMPDL3B has a strong correlation with immune cell infiltration in the tumor microenvironment of GAC, particularly the presence of M2 macrophages. In turn, M2 macrophages can also promote the proliferation of GAC cells. This study provides a reference for further research on SMPDL3B and its role in tumor diseases and offers some fresh concepts for the identification and GAC management.

## Data Availability

The data obtained and compiled for this work are not publicly accessible; however, they may be obtained from the corresponding author upon request.
